# Consistent and Correct Use of Condoms With Lubricants and Associated Factors Among Men Who Have Sex With Men from the Ghana Men’s Study II: Protocol for a Mixed Methods Study

**DOI:** 10.2196/63276

**Published:** 2024-12-03

**Authors:** Ratif Abdulai, Edith Phalane, Kyeremeh Atuahene, Refilwe Nancy Phaswana-Mafuya

**Affiliations:** 1 South African Medical Research Council/University of Johannesburg (SAMRC/UJ) Pan African Centre for Epidemics Research (PACER) Extramural Unit Johannesburg South Africa; 2 Department of Research, Monitoring, and Evaluation Ghana AIDS Commission Accra Ghana

**Keywords:** men who have sex with men, consistent condom use, HIV, Ghana Men’s Study, condom, Ghana, Africa, mixed methods, protocol, lubricant, qualitative, quantitative

## Abstract

**Background:**

Men who have sex with men (MSM) experience a disproportionate burden of HIV infection globally, including in Ghana. The use of condoms with lubricants correctly and consistently plays a vital role in reducing the number of new HIV infections among MSM. However, there are concerns about the consistent and correct use of condoms and lubricants among MSM in Ghana. In this regard, there is a need to understand context-specific factors associated with consistent and correct condom use with lubricants.

**Objective:**

This study aims to determine the current scope of consistent and correct use of condoms with lubricants, associated factors, interventions, and user- and service-related challenges on correct condom and lubricant use among the MSM population in Ghana.

**Methods:**

The study will use a mixed methods study approach. First, a retrospective analysis of the Ghana Men’s Study II data set involving 4095 MSM will be conducted to determine the scope of consistency and correct use of condoms with lubricants as well as associated factors. The data will be imported into STATA (version 17; StataCorp LLC) to treat missing data and outliers before the analysis. Bivariate and multivariate logistic regression analyses will be conducted to determine the associated factors of consistent condom use with lubricants. All statistical analyses will be done at a 95% CI, with significant differences at *P*<.05. Second, in-depth interviews with a purposive sample of about 15-20 stakeholders will also be conducted to understand contextual issues regarding the factors identified, identify existing interventions for correct condom and lubricant use, user and service-related challenges, and how best to address those challenges from the stakeholders’ perspectives. For qualitative data, thematic analysis will be conducted using Atlas.ti version 23.1.1.

**Results:**

Qualitative and quantitative results will be triangulated together with systematic review results, and key findings will be highlighted and used to guide the development of a predictive model for improving correct and consistent condom use with lubrication among MSM. This protocol paper, part of a doctoral study by the first author (RA), received approval from the Research and Ethics Committee of the University of Johannesburg on May 10, 2024. Data collection commenced on August 20, 2024, and the expected results will be published by October 2025.

**Conclusions:**

Results from qualitative interviews and secondary data analysis will be triangulated to develop a predictive model to strengthen the correct and consistent use of condoms with condom-compatible lubricants among MSM and other key population groups in Ghana and other parts of sub-Saharan Africa for future pandemic preparedness, policy making, and targeted budget allocation.

**International Registered Report Identifier (IRRID):**

DERR1-10.2196/63276

## Introduction

HIV infection is still an issue of public concern in sub-Saharan Africa, where many young adults engage in unprotected and transactional sexual relationships and also report inconsistent condom use [1]. Key populations (KPs), particularly men who have sex with men (MSM), female sex workers, people who inject drugs or people who use drugs, and transgender people, are disproportionally affected by the HIV burden [2]. In 2022, KPs and their sexual partners accounted for more than half (55%) of new HIV infections in sub-Saharan Africa [3]. Recent studies have shown that less effort has been made to reduce the incidence and prevalence of HIV in MSM in both underdeveloped and developed countries [4-6]. Men who have sex with men are more likely to contract and transmit HIV because they are more stigmatized and hidden, especially in low- and middle-income countries [7,8], aggravated by inadequate access to essential prevention materials such as condoms and lubricants [9].

In Ghana, HIV prevalence among MSM remains a major concern (18.1%) despite the remarkable decline in the number of new HIV infections among the general population (1.68%) [10]. The risk of HIV transmission in unprotected anal sexual activity is about 18 times higher than in unprotected vaginal sex [11,12], and 70% of this risk can be lessened by the consistent and correct use of condoms with lubricants [12]. According to model simulations, since 1990, the use of condoms consistently with lubricants has averted about 117 million new HIV infections [13,14]. Consistent use of condoms with lubricants to reduce the number of new HIV infections among MSM is a public health priority and an important component of prevention programs globally [11,12]. This is defined as using a new condom for each act of oral, anal, and vaginal sex during the whole sex act, ensuring that one uses ample lubricant during vaginal and anal sex, and removing the condom immediately after ejaculation to prevent spillage [11].

National estimates show that only 48.2% of MSM consistently and correctly use condoms during penetrative anal sexual intercourse with other male partners, compared with 29.7% of women [10]. This shows that consistently using condoms with condom-compatible lubricants (CCLs) correctly among MSM may be influenced by context-specific factors. Without proof of these factors, it will be impossible to address the underlying HIV prevention needs of MSM as well as other KP groups in Ghana. Further, there is inadequate consolidated data on the consistent use of condoms with CCLs and their associated factors among MSM in Ghana. This article therefore describes a protocol to determine the consistency of the correct use of condoms with CCLs and associated factors among MSM in Ghana as well as to identify existing interventions for correct condom and lubricant use, user and service-related challenges, and how best to address those challenges from the perspectives of the stakeholders. This study aims to determine the current scope of consistent condom use with lubricants and associated factors, as well as to develop a predictive model to improve the correct and consistent use of condoms with CCLs among MSM and other KP groups in Ghana, particularly for future pandemic preparedness, policy making, and funds allocations.

## Methods

### Overview

This is a mixed methods study involving retrospective analysis of secondary data and in-depth interviews with stakeholders. The reason for choosing a mixed methods study approach is that the secondary data analysis will help to determine the current scope of consistent and correct use of condoms with CCLs and associated factors among the MSM population in Ghana while interviews with stakeholders such as MSM, MSM representatives, program managers, and health service providers will also provide in-depth knowledge and understanding of the factors that will be identified during the secondary data analysis. The interviews will also help to provide useful information to address the challenges associated with the consistent and correct use of condoms with lubricants from the perspectives of both users and service providers.

### Methods for Retrospective Analysis of the Ghana Men’s Study II

#### Study Setting

The Ghana Men’s Study II was conducted in Ghana, located in the West African subregion, with 16 administrative regions and 261 districts [15]. The study was done in 2017 across the then 10 regions of Ghana. The population of Ghana has an estimated 55,000 MSM with 18.1% HIV prevalence compared with 1.6% in the general population [16-18].

The Ghana Men’s Study II was conducted in 2017 by the Ghana AIDS Commission (GAC), a supraministerial and multisectoral body established under the chairmanship of the president of the Republic of Ghana to provide HIV, tuberculosis, and sexually transmitted infections services to MSM, transgender, adolescents, young women, and other high-risk KP groups in Ghana, mainly supported by the Global Fund and the United States President’s Emergency Plan for AIDS Relief.

#### Study Design

A retrospective analysis of the Ghana Men’s Study II data set will be carried out to determine the scope of consistent and correct use of condoms with CCLs and associated sociodemographic, behavioral, socioeconomic, and structural factors among MSM.

#### Sample

The Ghana Men’s Study II included MSM who were biologically male, were aged ≤18 years (ie, inclusive of men aged 18 years), had consensual sex with another male partner in the last 12 months (self-reported), and lived, worked, or socialized in any one of the study regions of Ghana. Transgender women were eligible if they were biologically male and had intercourse with another male individual within the previous 12 months.

MSM were recruited using respondent-driven sampling, which is a widely used variant of snowball sampling where respondents are chosen not from a sampling frame but from a social network of existing participants of the sample. Incentives were provided for participation and the recruitment of others [19]. The size of the sample was determined based on the surveillance goal of following significant changes in the HIV epidemic over some time, that is, between rounds of the integrated biological and behavioral surveillance survey. The survey treated each of the sites as a distinct survey, with the size of the sample required to track any changes at each of the locations [10]. A sample size of 500 MSM for each region was calculated by factors such as the estimated period of the recruitment, funding, the highly risky nature of such studies, and, most importantly, the ability to measure the most important indicators using Ghana Men’s Study I estimates with 80% power at a 95% confidence level [10]. A total sample size of 4095 MSM were recruited for the Ghana Men’s Study II. The target sample generated an exact estimate of the anticipated equilibrium distribution for each of the indicators (change in sexual behavior, ie, the prevalence of always using condoms at the last sexual intercourse) to identify a 10%-15% increase (ie, from 48.4% to 53.4%) or decrease (28.4% to 23.4%) in the indicators based on the ability to measure significant program effort between surveys and HIV prevalence, with an expected projection of 34.3% [10]. For further details on the sample, please refer to earlier publications [10,20]

#### Data Collection

The primary outcome of the study is consistent condom use with lubricants at last sexual intercourse, measured as the number of participants with one or more intimate partners during the past 12 months (sexually active participants) [10]. The secondary outcomes are factors associated with consistent condom use with lubricants. The study will examine sociodemographic, behavioral, socioeconomic, and structural characteristics for the secondary outcomes. Data were collected on sociodemographics, condom and lubricant use, consistency of use, accessibility of condoms and lubricants, condom and lubricant use at last sex with a male or a female, and the consistency of condom and lubricant use (“always used a condom with lubricants” or “not always using condom with lubricants”) among MSM in Ghana during penetrative anal sex with other men, among others (Table 1). The GAC collected data through the MSM Comprehensive HIV Prevention Programme [10]. The questionnaire was administered through a computer, with respondents having the option of completing it using computer-assisted personal interview software [10]. The data will be secured from the GAC and used only for this study. The data were captured on the GAC’s electronic database and stored on a secure server with encryption and user access control. All the collected data were stored in a respondent-driven sampling-A-compatible format. The Bryant Research System was used to oversee and coordinate the collection of data to ensure anonymity, privacy, and confidentiality [10].

**Table 1:** Description of variables to be assessed from the 2017 Ghana Men’s Study II data set. Adopted from the Ghana Men’s Study II questionnaire [10].

Variable name			Variables		Variable descriptions		Variable recode
**Dependent variables (condoms and lubricants use)**							
	Condom use	Used a condom at last sexual intercourse		Yes No No response		Yes No	
	Lubricant use	Used a lubricant at last sexual intercourse		Yes No No response		Yes No	
	Condom use with lubricants	Used a condom with lubricants at last sexual intercourse		Yes No		Yes No	
	Consistent use of condoms with lubricants	Used condom with lubricants consistently “Always used a condom”, “frequent condom use”, and “consistently used a condom” are synonymous with condom use consistency during sexual activities		Yes No		Yes No	
	Consistent use of condoms with condom-compatible lubricants	Used condoms with condom-compatible lubricants consistently		Yes No		Yes No	
	Consistency of correct condom use with lubricants	Used condoms with lubricants correctly and consistently		Yes No		Yes No	
	Factors associated with condom and lubricant use	Positively associated factors of condom use Negatively associated factors of condom use		Sociodemographic factors Behavioral factors Economic factors Structural factors.		Sociodemographic factors Behavioral factors Economic factors Structural factors.	
**Factors that affect condom and lubricant use by classification: independent variables (sociodemographics)**							
	Age	Respondent’s age		Integer		≥18 years 20-24 years 25-29 years 30-34 years 35-39 years 40-44 years 45-49 years 50-54 years 55-59 years ≥ 60 years	
	Education	The educational level of the respondents		No formal education Basic education Secondary education Tertiary education		No formal education Basic education Secondary education Tertiary education	
	Occupation	The employment status of participants		Unemployed Self-employed Government employed Private-sector employed		Unemployed Self-employed Government employed Private-sector employed	
**Factors that affect condom and lubricant use by classification: independent variables (behavioral factors)**							
	Behavioral factors	Behaviors that affect condom and lubricant use		Number of sexual partners Condom-carrying Drug use or sex under the influence of drugs HIV testing behavior Trust for a sexual partner Seeking counseling on risk reduction. Dislike condom use Unplanned sexual intercourse		Number of sexual partners Condom-carrying Drug use or sex under the influence of drugs HIV testing behavior Trust for a sexual partner Seeking counseling on risk reduction Dislike condom use Unplanned sexual intercourse	
	Structural factors	Structural factors that influence condom and lubricant use		Physical and sexual violence Access challenges Low-quality products (eg, condoms) Distribution challenges Practical difficulties with using condoms such as breakage Misinformation about condom use Interference with sexual pleasure Access to free condoms		Physical and sexual violence Access challenges Low-quality products (eg, condoms) Distribution challenges Practical difficulties with using condoms such as breakage Misinformation about condom use Interference with sexual pleasure Access to free condoms	
	Socio-economic factors	Socio-economic factors affecting condoms and lubricant use		Financial incentives for sexual intercourse Socioeconomic vulnerability Financial stability		Financial incentives for sexual intercourse Socioeconomic vulnerability Financial stability	

### Data Analysis

The Ghana Men’s Study II data set will be imported into STATA (version 17; StataCorp LLC) for data cleaning and processing before conducting statistical analysis. The data will be disaggregated by sociodemographic characteristics, condom use, condom use at last sex, consistent condom use with a male or female sexual partner or both, consistent condom use during insertive or receptive anal sex, consistency of correct condom use with lubricants, consistency of the correct use of condoms with CCLs, and associated factors (sociodemographic, behavioral, structural, and socioeconomic factors) that promote or hinder condom and lubricant use consistency. Descriptive statistics will be performed to describe the demographic characteristics of MSM. Continuous variables will be reported as means with SD and categorical variables as percentages. We will conduct bivariate and multivariate logistic regression analyses to identify significant associations between consistent condom use with lubricants and sociodemographic factors, behavioral factors, and structural and socioeconomic factors. All statistical analyses will be done at a 95% CI, with significant differences at *P*<.05.

Concerning the choice of specific statistical test, one of the objectives of this study is to test for the associated factors of consistent condom use with lubricants hence our choice of Bivariate and Multivariate logistic regression analysis. After bivariate analysis, multivariate logistic regression analysis will be done to exclude all confounding factors to determine the factors that are associated with consistent and correct use of condoms with lubricants.

#### Methods for In-Depth Interviews With Stakeholders

##### Study Design

An exploratory study design of in-depth stakeholder interviews will be conducted, using a semistructured questionnaire (Multimedia Appendix 1).

#### Study Population

The stakeholders will comprise MSM and MSM representatives from various institutions who work closely with KPs, including the KP community, program managers, HIV health care service providers, and civil society organizations.

#### Sample

A purposive sample of about 15-20 key informants [20,21] of the various stakeholders that offer interventions, HIV prevention programs, and services to improve consistent and correct use of condoms and lubricants among MSM will be purposively selected to participate in the study. The sample size given is aimed at getting diverse views from a range of stakeholders and the number of key informants (15-20) is not fixed. It will increase until the saturation point is reached. Textbox 1 shows the inclusion and exclusion criteria.

**Inclusion and exclusion criteria for stakeholder participation in interviews on interventions for condoms and lubricant use [1].**

**Participants**

- Men who have sex with men (MSM), MSM representatives, HIV health care service providers, program managers, and civil society organizations

**Inclusion criteria**

- Individuals working in the HIV field as health care providers, program developers, policy makers, and beneficiaries aged 18 years and above
- They should be willing to provide informed consent to take part in the study
- They should have been working in the area of HIV control and prevention (ie, condom and lubricant use) for at least 1 year or more
- They should be employed in the field at the time of the interviews
- They should be knowledgeable on the subject matter
- They should speak English or the local language

**Exclusion criteria**

- Individuals younger than 18 years
- Individuals who did not provide informed consent to take part in the study
- Individuals working in the area of HIV control and prevention for less than a year
- Individuals who cannot speak English or the local language
- Individuals who are not knowledgeable on the subject matter
- Individuals who are not interested in the subject matter

### Participant Recruitment

The recruitment of stakeholders for this objective will be done with the support of the GAC, which has an extensive track record of working with MSM structures, leaders, and organizations. For MSM, the recruitment process will not be event-based recruitment however, a direct approach will be used to contact MSM individually. A known GAC staff member will approach potential MSM participants who were previously involved in GAC studies or activities. The GAC will provide an introductory support letter to each potential participant. If a potential participant agrees to participate in the study, they will be given the researcher’s contact details for engagement at a convenient time. The GAC will inform participants to feel free to contact them if they have any issues before, during, or after the study. The researcher will also be required to give feedback to GAC about any concerns or progress updates weekly in writing. The GAC will ensure that whatever concerns are raised by the participants are promptly addressed. The GAC will emphasize that participation in the study is voluntary and that participants may feel free to leave the study at any time. For the other stakeholders, in addition to the above-mentioned introductory letter, the researcher will write a letter of invitation to participate in the study. The invitation letter will specify the inclusion and exclusion criteria, especially emphasizing anonymity, privacy, confidentiality, and voluntary participation. The letter will specify that the interviews will not be focusing on their personal lives but rather on stakeholder views as officials working in the HIV field. This will be mentioned to ensure that participants are assured of protection against social, physical, or psychological vulnerabilities.

### Data Collection

The in-depth interviews will be conducted to elicit information on factors associated with condom use, the programs or initiatives and policies on the consistent and correct use of condoms and lubricants, user and service-related challenges, and how these challenges can be addressed among MSM and other stakeholders. Interviews will be administered face-to-face in a private room for participants’ privacy and will take place at a time and location that is most convenient for them. During face-to-face interview sessions, only the interviewer, interviewee, and note-taker will be present. During the interviews, only the interviewer (researcher), interviewee, and a trained note-taker will be present during all face-to-face interview sessions. For privacy purposes, a separate private room within their premises of choice will be used to conduct the interviews.

The interviews will commence with a brief introduction of the study objectives, introducing the interviewer, and reading the privacy declaration form. The participants will be asked to voluntarily engage in interview questions. The interviews will focus mainly on the study objective and probing questions will be used where necessary to get a deeper understanding of their views. Each interview is expected to last for about 45 minutes. No personal or identifiable information will be disclosed throughout the study The interviews will be recorded verbatim and supported by tape recording for accuracy purposes. Field notes will also be taken to supplement the recorded information. Data will be coded to generate themes and subthemes, where possible.

### Data Analysis

Data will be captured on Atlas.ti (version 23.1.1) and will be coded using an inductive approach. A comprehensive overview of the entire data will be done to become familiar with its content. Before analyzing the data, the interviews will be transcribed; thereafter, the entire dataset will be read to generate codes. Data will be coded using Atlas. ti and intercoder agreement will be used to deal with discrepancies. Patterns from created codes will be used to formulate themes and subthemes where necessary. After this, a consensus on the themes and subthemes will be reached before further analysis. Finally, themes will be defined and named; then, the data analysis will be carried out. Thematic content analysis will be used to analyze open-ended questions, whereas closed-ended questions will be analyzed descriptively, reporting proportions and percentages.

### Ethical Considerations

This study protocol will use deidentified data from the Ghana Men’s Study II dataset secured from the GAC. After the data analyses, the preliminary results will be stored securely on a memory stick with an access code. The results of this study will only be reviewed by the researcher and the research team. All the data used in this study will be managed in compliance with the Protection of Public Information Act. Furthermore, all the data generated from the PhD study will be stored for 5 years after the completion of the study.

The results to be published will not have any identifiable information. This protocol paper is part of the doctoral study of the first author (RA) and has received ethics approval from the Research and Ethics Committee (REC-2742-2024) (Multimedia Appendix 2) of the University of Johannesburg. Furthermore, this study also falls under a broader research project at the South African Medical Research Council (SAMRC)/the University of Johannesburg (UJ) Pan African Centre for Epidemic Research (PACER) Extramural Unit funded project, namely Harnessing Big Heterogeneous Data to Evaluate the Potential Impact of HIV Responses Among KPs in Generalized Epidemic Settings in Sub-Saharan Africa (REC-1504-2023).

Before obtaining consent, the study participants will be provided with the full research details before participating in the qualitative study. These include the objectives, methods, anticipated benefits, and possible risks of the research. Written informed consent will be taken in a private room at a convenient time, and location, and on an individual basis with the researcher and the note taker. The content of the consent form will be read out to the participants and ensure that they understand and ask for their consent. The decision to participate will solely rest on the participant and no form of coercion, deceit, or influence will be used on them. Participants will be informed that their participation in the study is voluntary and that they have the right to withdraw from the study if they so choose at any moment, for any reason, and without any repercussions or prejudice. There will be contact information for the researcher and the supervisor available should any questions or concerns arise about the research and the research participant’s rights. This study will only involve individuals with a complete willingness to participate. Before participation, all participants will be asked to sign the informed consent form, protection of public information consent for the collection of information, and consent to audiotape the interviews to confirm their complete willingness to participate in the study.

To ensure the confidentiality of the study participants, a private room will be used for the interview, and the interview will be conducted at a time and location suitable to participants. During interviews, only the interviewer, interviewee, and trained note-taker will be present during all face-to-face sessions. The note-taker will sign a confidentiality agreement to ensure that no information is divulged without the permission of the participant. We will ensure that pseudonyms are used for each participant and that no identifiable information will be found in the interview transcripts. Steps will be taken to control data accessibility and all interview recordings and paper-based data collection materials will be secured under lock and key.

For participants’ safety, the study will be entirely anonymous; at no point or time will names or identifying information of the MSM be collected. The GAC, which has an extensive track record of working with MSM structures and leaders, will lead the recruitment process. As outlined in the participants’ recruitment section, there will be no organized meetings with MSM groups to avoid any risk of exposure. There will be no campaigns for MSM to join the study and no social marketing of the study. The use of any type of media, including TV and radio advertisements, will be avoided. A direct approach will be used to contact MSM individually. A known GAC staff member will approach potential MSM participants previously involved in GAC studies or activities. The GAC will provide an introductory support letter to each potential participant. If a potential participant agrees to participate in the study, they will be given the researcher’s contact details for engagement at a convenient time. The GAC will inform participants to feel free to contact them if they have any issues before, during, or after the study. The researcher will also be required to give feedback to GAC about any concerns or progress updates weekly in writing. The GAC will ensure that whatever concerns are raised by the participants are promptly addressed.

In addition, there is also a trust relationship between the GAC and MSM that has been nurtured and grown for over 2 decades of HIV programming among MSM who receive the full range of HIV services in all 16 regions of the country. The program is founded on public health and human rights principles with well-established protective mechanisms, developed and implemented in strong collaboration with the Ghana Police Service and other law enforcement agencies. As part of this collaboration, the Ghana Police Service adopted and integrated public health and human rights principles into its pre- and in-service training curricula to prepare the personnel for the effective protection of KPs. The police service also established national and regional steering committees to coordinate and oversee the protection of KPs, including MSM throughout the country. A special unit of the police service with officers trained in public health and human rights principles handles all MSM-related cases in the country just to ensure their protection. The protection efforts of the police service are complemented by trained paralegals and pro-bono lawyers who work closely with the police to ensure the protection and uphold of the dignity of MSM who find themselves on the wrong side of the law. The strong partnership and collaboration with the police service will be used to as well protect any study participant who may be involved in any adverse incident.

## Results

The results will encompass analysis of deidentified data from the Ghana Men’s Study II dataset, as well as in-depth stakeholder interviews, to determine the current scope of the consistency of correct condom use with lubricants and the associated factors among the MSM population in Ghana. Results from the secondary data analysis will be presented in tables and interpreted by using odds ratios and *P* values. For the qualitative interviews, we will do interpretation by making a list of key themes and subthemes. Each theme that arises during the coding process will be reviewed to identify similarities and differences in responses from participants. Relationships between themes to determine how they are connected will be considered. Key findings will be highlighted and used to guide the development of a predictive model for improving correct and consistent condom use with lubricants among MSM. This protocol paper is part of a doctoral study by the first author (RA), which received approval from the Research and Ethics Committee of the University of Johannesburg on May 10, 2024. Secondary data analysis commenced on August 20, 2024, and all expected results will be published by October 2025.

## Discussion

### Principal Findings

Consistency of the correct use of condoms with CCLs is very crucial in preventing new HIV infections among MSM and other KP groups in Ghana and across the globe. The study will use a mixed methods study design, retrospective data analysis, and stakeholder interviews. The rationale for this approach is that the secondary data analysis will elicit information on the scope of consistent and correct condom use with CCLs and associated factors among MSM while interviews with the stakeholders will also provide in-depth knowledge and understanding of the factors that will be identified during the secondary data analysis. The interviews will also help to provide useful information to address the challenges associated with the consistent and correct use of condoms with lubricants from the perspectives of both users and service providers. Findings from the study will be triangulated to develop a predictive model to improve the consistency of the correct use of condoms with lubricants among MSM and other KP groups [22-25]. This study corroborates the findings of another study conducted by Newby et al [26], which proposed the development of a future-definitive trial to reduce the occurrence of sexually transmitted infections by improving the correct and consistent use of condoms.

Results from this study will help to address a significant gap in understanding sexual health practices among the MSM population in Ghana. This study will also provide useful information essential for developing targeted interventions and policies to mitigate the impact of HIV on public health. By using predictive modeling techniques, this study has the potential to increase awareness and promote safer sex practices among MSM, ultimately informing evidence-based strategies for HIV prevention and control.

The monitoring and evaluating HIV prevention framework and the SMART (specific, measurable, attainable, realistic, and timely) tool will serve as a guide for developing the model. The SMART tool allows users to capture, manage, and map data from studies or surveys that can be analyzed to understand behavioral changes over time as well as understand the factors responsible for the changes. Positively associated sociodemographic factors, behavioral factors, structural factors, and socioeconomic factors of consistent condom use with lubricants will be triangulated to develop the model.

Regarding the dissemination plan, we intend to publish the study findings for each objective in a peer-reviewed journal with a high impact factor and readership by October 2025. A report summarizing the findings will be made available to stakeholders, policy makers, and relevant organizations to ensure that the correct and consistent use of condoms with CCLs is widely practiced among all KP groups and the general public.

### Strengths and Limitations

The retrospective analysis being undertaken relies on data from the Ghana Men’s Study II, which uses rigorous scientific methods successfully used in the Ghana Men’s Study I to ensure scientific rigor, comparability, and consistency. For example, a sample size calculation was done to ensure that the study sample was large enough to enable the generalization of the study results to the MSM population. The sample size was calculated based on the estimated MSM population size in Ghana. Multiple methods were used to recruit MSM to ensure that diverse MSM groups are represented in the study, thus enhancing the applicability of the study’s findings to MSM broadly. The same validated tools used in Ghana Men’s Study I were used in Ghana Men’s Study II to ensure the reliability and validity of the study results. Researchers in the Ghana Men’s Study received training to enable them to elicit information; however, subjectivity bias cannot be ruled out. Data collection tools were administered in local languages to ensure a proper understanding of the questions. The possibility of information bias may not be ruled out given that self-reported information was used.

In addition, qualitative interviews will be used to elicit rich, in-depth information on some of the findings emanating from the secondary data analysis. Further, a systematic review will be conducted to understand the status quo. The secondary data, qualitative data, and narrative analysis from the systematic review will be triangulated. The use of a mixed methods approach will mitigate the inherent limitations of a retrospective study design.

### Conclusion

This study will provide data to inform the design of a predictive model through stakeholder interviews and secondary data analysis to strengthen the correct and consistent use of condoms with CCLs among MSM and other KP groups in Ghana and other parts of sub-Saharan Africa. The outcome of this study could also inform the government of Ghana how best to support HIV prevention programs to meet the health care needs of KP groups in Ghana. This will enable the government of Ghana to reach its health goals, conforming to the third goal of the Sustainable Development Goals.

## Appendix

Multimedia Appendix 1 Interview guide.

Multimedia Appendix 2 Research and ethics approval letter.

## Abbreviations

CCL: condom-compatible lubricant
GAC: Ghana AIDS Commission
IBBS: integrated biological and behavioral surveillance survey
KP: key population
MSM: men who have sex with men
PACER: Pan African Centre for Epidemics Research
SAMRC: South African Medical Research Council
UJ: University of Johannesburg

## Data Availability

Data sharing is not applicable to this article as no data sets were generated or analyzed during this study.
