# Supplementary material for: Consistent and Correct Use of Condoms With Lubricants and Associated Factors Among Men Who Have Sex With Men from the Ghana Men’s Study II: Protocol for a Mixed Methods Study
Source: JMIR Res Protoc. 2024 Dec 3;13:e63276. doi: 10.2196/63276 (PMC11653033; doi:10.2196/63276)
Supplement: Multimedia Appendix 1 [file resprot_v13i1e63276_app1.docx]

A Guide for In-depth Interviews with HIV Stakeholders

| QUESTION AND PROBES | NOTES/RATIONALE |
| --- | --- |
| **Background/icebreakers** |  |
| I would like to begin by asking some questions about you, your job, and what you do from day to day:   - Please tell me a bit about yourself (Probes: locality, age, gender, duration of employment, your roles, etc | This section is designed to help the respondent feel at ease and to get to know the participant a little better. |
| Interventions (both ineffective and effective ones), policies, and programs on condom and lubricant use, user and other service-related challenges, and how best to address the challenges from stakeholders’ perspectives. |  |
| **1. Men who have sex with men**  Could we discuss HIV prevention interventions, policies, and programs for the correct and consistent use of condoms and lubricants?   - What type (s) of HIV prevention interventions, policies, or programs on condoms and lubricants use do you know? - Which one (s) of the prevention interventions, policies, or programs on condoms and lubricants use do you think is/are most effective and why? Please elaborate…. - Which one (s) is/are ineffective and why do you think that they are no longer fit for purpose? - What types of condoms and lubricants do you know are available? - What do you think about the protective nature of condoms especially when used with lubricants? Please elaborate ……. - Can you tell me how you understand consistent and correct use of condoms and lubricants? - Do you prefer to use condoms together with lubricant? If not, please share with me your reason(s). if yes, how consistently do you use them? - Do you easily have access to condoms and lubricants? If not, tell me tell challenges and how best we can resolve them. - What factors hinder the distribution and accessibility of condoms and lubricants? - What possible available routes do you use to reach out to MSM? - How best can we improve access among MSM? | To understand what factors are associated with condoms and lubricant distribution and uptake as well as consistency and correct use and how best to address challenges from the stakeholder’s viewpoint |
| **HIV Healthcare Service providers, MSM implementers Program managers, and CSOs.**  1 HIV prevention interventions   - Do you have any HIV prevention intervention program in place? - If no, what do you plan to do to help prevent the further spread of HIV among MSM? - Any reasons why you do not have any HIV prevention intervention program in place? - If yes, what are some of the HIV prevention interventions that you have in place? - Which of the interventions is/are more effective and why? - What do you think accounts for the ineffectiveness of other interventions? - What are the major challenges that you are faced with? - Share your view on how best these challenges can be addressed. | To identify and understand some of the challenges that hinder the effectiveness and performance of HIV prevention interventions and address them through the stakeholder’s point of view. |
| **Recommendations** |  |
| Finally, I would like your recommendations to help improve the distribution, uptake, and correct and  consistent use of condoms and lubricants. | To understand what they feel are priorities for change in health services. |
| Wrap up   - Is there anything else that you would like to discuss today? - Are there any questions you would like to ask me about anything we have discussed today? |  |
