# Supplementary material for: Consistent and Correct Use of Condoms With Lubricants and Associated Factors Among Men Who Have Sex With Men from the Ghana Men’s Study II: Protocol for a Mixed Methods Study
Source: JMIR Res Protoc. 2024 Dec 3;13:e63276. doi: 10.2196/63276 (PMC11653033; doi:10.2196/63276)
Supplement: Multimedia Appendix 2 [file resprot_v13i1e63276_app2.pdf]

FACULTY OF HEALTH SCIENCES
RESEARCH ETHICS COMMITTEE

NHREC Registration: REC 241112-035

ETHICAL CLEARANCE LETTER
(RECX 2.0)

Student/Researcher Name	Ratif Abdulai	Student Number	223052905
Supervisor Name	Phaswana-Mafuya, Metse		
Department	Environmental Health		
Research Title	CONDOM AND LUBRICANT DISTRIBUTION, CONSISTENCY OF CORRECT USE AND ASSOCIATED FACTORS AMONG MEN WHO HAVE SEX WITH MEN IN GHANA – A PREDICTIVE MODEL FOR ADDRESSING COVID-19 IMPACTS		
Date	10 May 2024	Clearance Number	**REC-2742-2024**

Approval of the research proposal with details given above is granted, subject to any conditions under 1 below, and is valid until 2025/05/09.

1. Conditions:
Gatekeeper permission, as required. Local research ethics approval in the country where the research is conducted prior to data collection (approval letter to be submitted to the FHS REC). Reporting of adverse events to FHS REC.
**Please note that failure to comply with the conditions above (if any) prior to implementation of the research will invalidate this ethical clearance.*

2. Renewal:
It is required that this ethical clearance is renewed annually, within two weeks of the date indicated above. Renewal must be done using the Ethical Clearance Renewal Form (REC 10.0), to be completed and submitted to the Faculty Administration office. *See Section 12 of the REC Standard Operating Procedures*.

3. Amendments:
Any envisaged amendments to the research proposal that has been granted ethical clearance must be submitted to the REC using the Research Proposal Amendment Application Form (REC 8.0) prior to the research being amended. Amendments to research may only be carried out once a new ethical clearance letter is issued. *See Section 13 of the REC Standard Operating Procedures*.

4. Adverse Events, Deviations or Non-compliance:
Adverse events, research proposal deviations or non-compliance must be reported within the stipulated time-frames using the Adverse Event Reporting Form (REC 9.0). *See Section 14 of the REC Standard Operating Procedures*.

The REC wishes you all the best for your studies.

Yours sincerely.

Prof. Christopher Stein
Chairperson: REC
Tel: 011 559 6564
Email: cstein@uj.ac.za

Secretariat: Ms Raihaanah Pieterse
Tel: 011 559 6073 email: rpieterse@uj.ac.za

Secretariat: Ms Raihaanah Pieterse
Tel: 011 559 6073 email: rpieterse@uj.ac.za
